# Green Reduction of Graphene Oxide using Kaffir Lime Peel Extract (*Citrus hystrix)* and Its Application as Adsorbent for Methylene Blue

**DOI:** 10.1038/s41598-020-57433-9

**Published:** 2020-01-20

**Authors:** Ronald Wijaya, Gracia Andersan, Shella Permatasari Santoso, Wenny Irawaty

**Affiliations:** 1 Department of Chemical Engineering, Widya Mandala Surabaya Catholic University, Kalijudan 37, Surabaya, 60114 Indonesia; 2 Department of Chemical Engineering, National Taiwan University of Science and Technology, No. 43, Sec. 4, Keelung Rd, Da’an District, Taipei City, Taiwan

**Keywords:** Synthesis of graphene, Environmental impact

## Abstract

Green reduction of graphene oxide (GO) by phytochemicals was explored using the aqueous extract of kaffir lime peels. The research methods included preparation of extracts, preparation of GO, preparation and characterization of reduced-GO (RGO) using Fourier Transform Infrared (FTIR), X-ray diffraction (XRD), and UV-Vis spectroscopy, as well as methylene blue (MB) adsorption test using RGO. The RGO characterization showed that GO was successfully reduced by a C=C group restoration. The MB adsorption kinetics profile in RGO is more suitable for the pseudo-second-order model, whereas for the adsorption isotherm it is more suitable for the Langmuir model with a maximum adsorption capacity (q_max_) of 276.06 mg/g at room temperature. The best ratio of GO: kaffir lime peel extract used to prepare RGO was at a ratio of 1: 2. Based on the ΔG, ΔH, and ΔS values, the adsorption of RGO-MB was defined as spontaneous and endothermic process. The results promise the potential application of RGO derived via green route to remove cationic dye in wastewater.

## Introduction

Methylene Blue (MB) is a cationic dye that has a dark green color^1^. MB is very soluble in water and alcohol and often used as the coloring agent for textiles^2^. Methylene blue can have a negative impact on human body^3^. If inhaled, it can cause respiratory disease, when MB is accidentally swallowed, it will produce a burning sensation and can cause nausea, vomiting, diarrhea, gastritis, abdominal and chest pain, severe headaches, lots of sweating, mental confusion and methemoglobinemia^4^. Therefore, the presence of MB in the water needs to be well addressed.

There are several methods used to treat waste from textile water pollution, by ultrafiltration method^5^, ozonization^6^, ion exchange^7^, photocatalysis^8^, and adsorption^9^. Of the many methods, adsorption the most promising method for dealing with textile wastewater; using commercial adsorbents such as activated carbon^10^, zeolites^11^ and nanomagnetic materials^11–13^. In this study, the concentration of MB in simulated textile wastewater was reduced by the adsorption onto the surface of graphene oxide-based material.

Graphene Oxide (GO) is a carbon nanomaterial that can make strong interaction with organic molecules through hydrogen bonds and π-π interaction^14^. GO has a stable 2-dimensional structure as electrical and thermal conductors^15^. GO is widely used in many applications such as sensor^16^, catalyst^17^, electronics^18^, water splitting^19^, CO_2_ reduction^20^, and water treatment^17,21^. The ability of GO to adsorb MB is tremendous, with removal efficiency up to 98.8%^14^. Some modifications can be applied to enhance the ability of GO in MB adsorption, such as composite photocatalyst^17,22,23^. A further modification to improve the performance of GO-based materials is essential. GO is heavily decorated by the oxygen functional group (C-O), and therefore if the oxygen groups can be reduced, the surface area may be expanded. In this state, GO is turned into Reduced Graphene Oxide (RGO).

The conventional method to reduce GO is performed using chemicals such as hydrazine^24^, sodium borohydride^25^, hydroxylamine^26^, etc. The chemical synthesis route offers an efficient method, but the chemicals used are not environmentally friendly. The decrease of RGO performance due to the employment of strong reducing agent during the process has been reported^27^. Thermal treatment is another method to reduce GO^28,29^. The heat treatment can efficiently remove the oxygen-containing functional groups and thus, open the possibility to fabricate RGO. However, the system requires a high amount of energy as well as complicated experimental set-up. Electrochemical reduction offers faster and safer route compared to the previous methods, but its reduction efficiency is reported less than the hydrazine reduction^30^.

Among those methods, the reduction using chemical agents is still the most effective technique for producing RGO. However, the use of hazardous chemicals during RGO can be a problem for the environment^27^. Some natural compounds have been introduced to replace the use of the hazardous reducing agent, i.e. extract of eucalyptus leaves^31^, ascorbic acid^32^, green tea^33^, lemon^34^, etc. However, most bioreducing agents used in RGO preparation are food sources; this raises the possibility of food competition. Therefore, there is still a need to explore non-food sources as reducing agents; agricultural by-products such as peels, seeds, etc, can be a potential source. Herein, we present the RGO preparation using a reducing agent prepared from kaffir lime peel. The reduction of GO using kaffir lime peel extract has not been reported in any literature. Our approach offers several advantages such as eco-friendly reducing agent, simple extract preparation, and non-toxic wastes generated at the end of reduction process.

## Materials and Methods

### Materials

Natural graphite powder was supplied by PT. Brataco (Surabaya, Indonesia). Hydrochloric acid (SAP, 37%), sulfuric acid (H_2_SO_4_, CAS 7664-93-9, 99.999%), hydrogen peroxide (H_2_O_2_, SAP, 30%), potassium permanganate (KMnO_4_, SAP, ≥99.0%), and methylene blue (C_16_H_18_ClN_3_S, CAS 122965-43-9) were used as received. Kaffir lime fruits were obtained from local kaffir lime plantation in Lumajang (East Java, Indonesia).

### Preparation of graphene oxide (GO)

GO was prepared by using the modified Hummer’s method^18^. The preparation procedure was briefly given as follows: 3 g of graphite powder was put into 70 mL of H_2_SO_4_ solution under ice bath conditions with the temperature was set below 20 °C, then 9 g of KMnO4 was added to the mixture. Subsequently, the mixture was transferred to an oil bath (Memmert), and the temperature was maintained at 40 °C. During the reaction process, the mixture was continuously stirred at 600 rpm for 30 min. When the reaction was completed, 150 ml of RO water was added, and the mixture constantly stirred at 600 rpm for 15 min within 95 °C. Then, 500 mL of water was added, followed by slow addition of 15 mL of H_2_O_2_ (30%) to remove the residue. A change of solution color was observed from brown to yellowish. The suspension is then filtered and washed with 250 mL of dilute HCl solution (1:10). The resulting solid was dried naturally, followed by adding 600 mL of water. The GO solution was then repeatedly washed with water to remove all residual salts and acids. The neutralized GO solution was stirred overnight, followed by the sonication process to exfoliate the graphite oxide into graphene oxide. The GO dispersion was then stored for the reduction process.

### Preparation of kaffir lime peel extract (Citrus hystrix)

Twenty-five grams of kaffir lime peels were extracted with 1000 mL ethanol (41%) at room temperature for 8 h. The extract was then filtered to separate the solid part and kept in closed container to be further used for GO reduction. Total Phenolic Content (TPC) test was carried out to determine the amount of phenolics in the extract of kaffir lime peels.

### Reduction of GO by kaffir lime peel extract (Cytrus hystrix)

GO and kaffir lime peel extract was mixed with the GO/extract ratio (v/v) 1:1, 1:2, and 1:4 under vigorous stirring (600 rpm) for 8 h. Subsequently, the mixtures were washed and sonicated for 30 min. This treatment was repeated several times until a clear solution was obtained. Then, the materials were dried in a vacuum oven (Vacucell, 55).

### Characterization

Ultraviolet-visible (UV-vis) spectroscopy of GO and RGO was recorded on a Shimadzu UVMINI-1240 spectrophotometer. Crystalline properties of graphite, GO, and RGO were analyzed using X-Ray Diffraction (XRD, Philips X’pert Xray Diffractometer) operated at 40 kV; 30 mA using Cu-Kα1 (λ = 0.15406) at 0.02° step size and 10.16 s step time from 5° to 60°. The surface functional group analysis of the graphite, GO, and RGO was analyzed using Fourier Transform Infrared (FTIR, Shimadzu 8400 S) using KBr powder method. The FTIR spectra were obtained at wavenumber from 1000 to 4000 cm^−1^.

### Preparation of methylene blue (MB) solutions

A stock solution of 1000 mg/L MB was made from MB powder, which was dissolved in water. The solution used in the experiment was prepared by diluting the stock solution from MB to 50 mg/L. The pH of the solution measured using pH meter (Metler Toledo).

### The experiment of MB adsorption

The contact time effect on MB adsorption was carried out by adding 5 mg RGO into 20 mL of MB solution with a concentration of 50 mg/L in a 100 mL erlenmeyer flask. The flasks were placed in a water bath shaker at 30 °C (Memmert SV-1422) and shaken until the equilibrium time was reached. At the specified time, the RGO and MB were separated by centrifugation at 4500 rpm for 10 min.

The adsorption isotherm experiment was conducted at room temperature using three variations of RGO (1:1, 1:2, and 1:4). The experiment was carried by different addition mass of adsorbent (2–12 mg, step size: 2 mg) into a series of erlenmeyer flasks, where each flask contains 20 mL of MB solution with a concentration of 50 mg/L. These flasks were shaken at 90 rpm until the equilibrium state was achieved. The RGO and MB were separated by centrifugation at 4500 rpm for 10 min. The final concentration of MB was analyzed by UV-Vis spectrophotometer at 665 nm.

The adsorption capacity of RGO was calculated by:

1 $${{\rm{q}}}_{{\rm{t}}}=(\frac{{{\rm{C}}}_{0}-{{\rm{C}}}_{{\rm{t}}}}{{\rm{m}}})\times V,$$

where *C*_*o*_ (mg/L) is an initial concentration of MB, *C*_*t*_ (mg/L) is the concentration of MB at time *t*, *m* (g) adsorbent quantity, and *V* (L) is the volume of MB solution. At a constant value of *C*_*t*_, the parameter of *C*_*t*_ and *q*_*t*_ becomes *C*_*e*_ and *q*_*e*_, where *C*_*e*_ (mg/L) is the equilibrium concentration of MB.

## Results and Discussion

### Characterization of graphene materials

**Figure 1:**
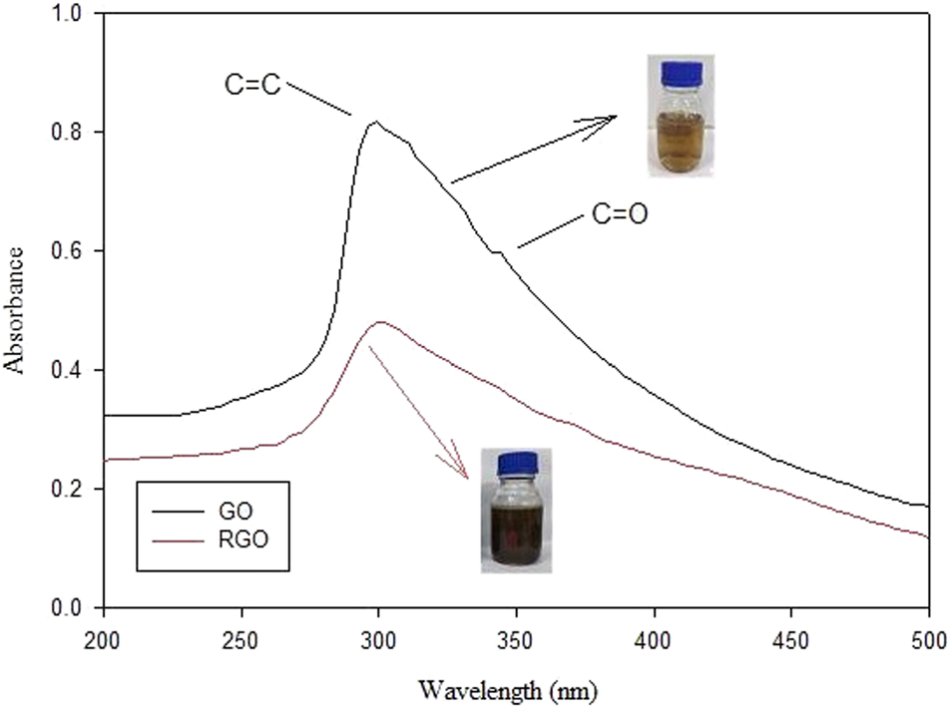
UV-Vis Spectrum of GO and RGO.

UV-Vis spectroscopic analysis was carried out to monitor changes in functional groups from GO to RGO. It can be seen from Fig. 1 that the peak transition π → π* for graphitic groups on GO was detected at a wavelength of ~300 nm. The shoulder peak at ~343 nm representing n → π* for the C=O group, indicates the presence of a C-O functional group in GO^32^. The GO transformation into RGO was confirmed by loss of the shoulder peak, where the C-O group in RGO has been reduced. The transformation of GO to RGO was also supported by the change of GO solution color, which was initially brown to blackish after being reduced by kaffir lime peels extract.

**Figure 2:**
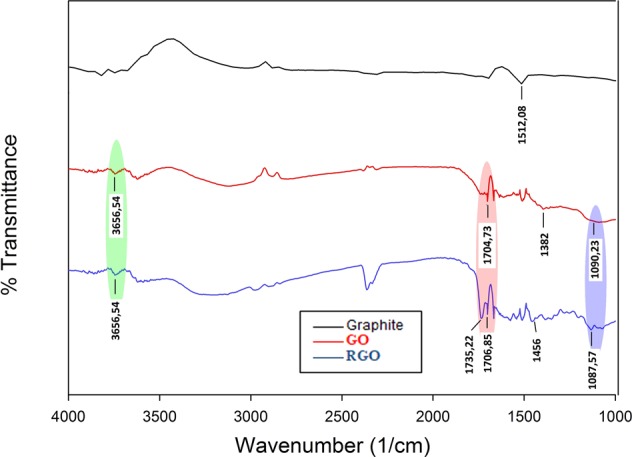
FT-IR spectra of graphite, GO, and RGO.

The surface functional groups of graphite, GO, and RGO were obtained by Fourier Transform Infrared (FTIR) analysis. Fig. 2 shows the presence of several functional groups in the three samples tested. The results of FTIR analysis summarized in Table 1 showed the presence of C=C groups in graphite at the peak of 1512.08 cm^−1^. The formation of GO was indicated by the presence of a hydroxyl group at 3656.54 cm^−1^, a carbonyl group (C=O) at 1704.73 cm^−1^, an alkene group (=CH) at 1090.23 cm^−1^, and ester group (C-O) at 1382 cm^−1^. The GO reduction process into RGO caused the aromatic group (C=C) restored by the emergence of 1456 cm^−1^ and 1735.22 cm^−1^ peaks in RGO. The change of the peak indicates that the GO was successfully reduced to RGO^35^. FTIR analysis shows that there are still C=O groups remained on the reduced GO; this signifies that the GO reduction partially occurred. However, it is still unclear as to whether C=O persists even after suffering a reduction. A similar phenomenon was experienced by Aliyev *et al*., where the RGO produced still has C=O groups remaining even after reduction using hydrazine monohydrate^36^. Dreyer and group suggest that in the process of GO reduction, the reducing agent can breakdown the epoxy groups of GO and produce carbonyl groups^37^.

**Table 1:** The surface functional groups of Graphite, GO and RGO.

Functional Group	Wavenumber (1/cm)		
	Graphite	GO	RGO
Alkane C-C	1502.73	—	—
Hydroxyl (O-H)	—	3656.54	3656.54
Carbonyl (C=O)	—	1704.73	1706.85
Alkene (=C-H)	—	1090.23	1087.57
Ester (C-O)	—	1382	—
Aromatic (C=C)	—	—	1456
Aromatic (C=C)	—	—	1735.22

**Figure 3:**
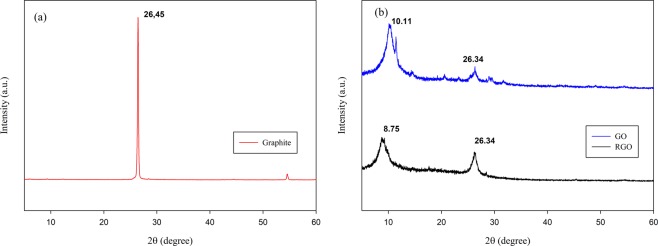
XRD patterns of (**a**) graphite (**b**) GO and RGO.

The X-Ray Diffraction (XRD) analysis was conducted to determine the crystallinity of graphite, GO, and RGO. The peak point of the GO is determined based on the degree of oxidation and the exfoliation process. It can be seen from Fig. 3 and Table 2 that graphite, which initially had a sharp graphitic peak at 26.45° and spacing of 3.35 Ǻ. After being synthesized became GO, the graphitic peak in 26.45° was found disappear, and new peak appeared at 10.11° with lots of diffraction. This indicates that synthesized graphite into GO undergoes peak shifting due to the oxidation and intercalation process of epoxy, hydroxyl, etc.^32^. The spacing or distance between layers that were initially 3.37 Ǻ changed to 8.75 Ǻ also indicates an increasing degree of oxidation, and the GO exfoliation process is getting stronger^38^. After being reduced being RGO it was found that the peak point in the area of 10.11° has broadened, and the graphitic peak intensity at 26.34° is increasing. Thus it can be indicated that the reduction process, there is a degradation in C-O groups and the restoration of the C=C bond structure.

**Figure 4:**
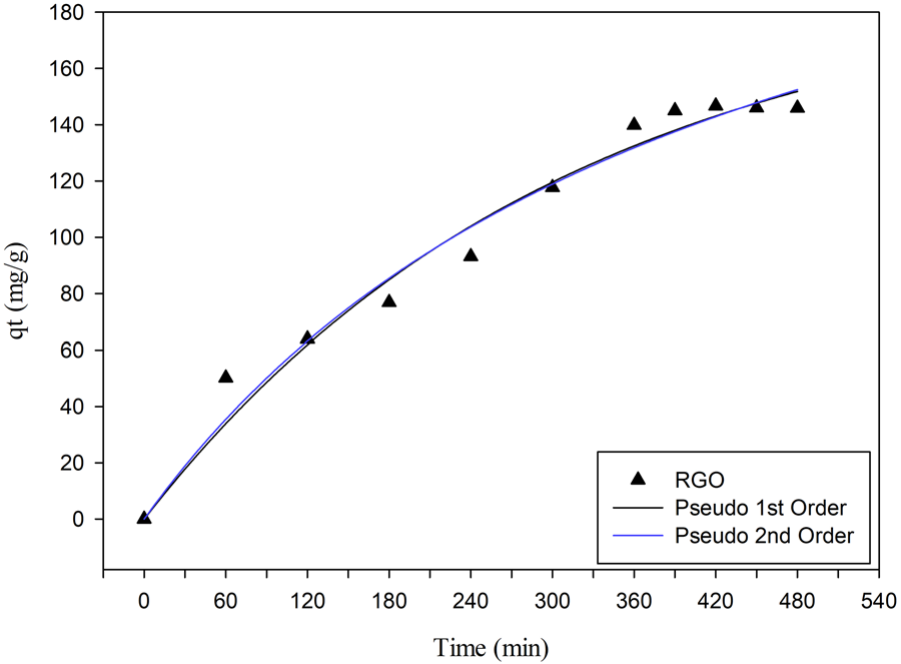
Adsorption kinetics of MB adsorption onto RGO.

**Table 2:** Peak result of XRD pattern for Graphite, GO, and RGO.

Sample	2ϴ (degree)	Spacing-d (Ǻ)
Graphite	26.45	3.37
GO	10.11	8.75
RGO	8.75	10.10
	26.34	3.38

### Adsorption kinetic study

The adsorption kinetic curve of MB over RGO are shown in Fig. 4. It can be seen that the adsorption equilibrium time was reached after 420 min. The adsorption kinetics parameter was determined using the pseudo-first and the pseudo-second-order models. These models are calculated using the following equations:

2 $$pseudo\, \mbox{-} \,first\,\log (qe-qt)=\,\log \,qe-\frac{{k}_{1}}{2.303}t$$

3 $$pseudo\, \mbox{-} \,second\,\frac{t}{qt}=\frac{1}{{k}_{2}\,q{e}^{2}}+\frac{t}{qe}$$

The parameters of the adsorption kinetics of MB over RGO are summarized in Table 3. Based on the sum square error (SSE), physisorption is the dominant process controlled the adsorption of MB on RGO. In reverse, if the model obtained refers to pseudo-second-order, then RGO-MB will tend to the mechanism of chemisorption^39^. The equilibrium state of MB adsorption on RGO obtained in kinetic experiment is used as reference time in adsorption isotherm process.

**Table 3:** Parameters of the pseudo-first-order and pseudo-second-order kinetic model for MB adsorption.

Kinetic Model	Parameters	Value
Pseudo-first-order	*qe* (mg/g)	192.79
	*k*_1_ (1/min)	0.0032
	SSE	606.53
Pseudo-second-order	*qe* (mg/g)	288.11
	*k*_2_ (g/mg min) × 10^−6^	8.12
	SSE	582.92

From the pseudo-first-order model, the *qe* value is 192.79 mg/g, and the *k*_1_ value is 0.0032 min^−1^ with SSE of 606.53. Meanwhile, the pseudo-second-order equation obtained *qe* of 288.11 mg/g and *k*_2_ value of 8.12 ((g/mg min) × 10^−6^) with SSE of 582.92. From the comparison of SSE values in the kinetic model used, the RGO adsorption kinetics is more in line with the pseudo-second-order, which means that the adsorption process of RGO-MB was more controlled by chemisorption.

### Isotherm adsorption study

The adsorption isotherm of RGO was carried out to study the interaction between adsorbate and adsorbent. Adsorption isotherm was carried out with a span of 420 min, where this time is equilibrium time as obtained from the adsorption kinetics data. The adsorption isotherm was modeled using the Langmuir and Freundlich models. The Freundlich and Langmuir model can be represented mathematically as follows:

4 $$Freundlich\,{q}_{e}={K}_{f}{{C}_{e}}^{1/n}$$

5 $$Langmuir\,{q}_{e}={q}_{max}\frac{({K}_{L}Ce)}{1+{K}_{L}Ce}$$

**Figure 5:**
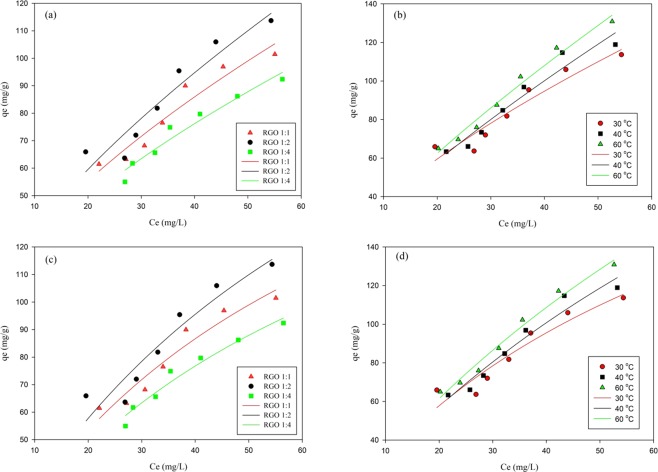
Adsorption isotherm of MB adsorption onto RGO. (**a**) Freundlich model at different ratio of GO/extract (**b**) Freundlich model at different temperatures (**c**) Langmuir model at different ratio of GO/extract (**d**) Langmuir model at different temperatures.

In the Freundlich model, *K*_*f*_ represents the adsorption capacity of the adsorbent, and *n* showed the heterogeneity and adsorption intensity of RGO. In the Langmuir model, *q*_*max*_ represents the maximum adsorption capacity, *K*_*L*_ states the Langmuir constant. The result of isotherm adsorption of RGO-MB is presented in Fig. 5a and Fig. 5c, and the calculated parameters are summarized in Table 4.

**Table 4:** Parameters of Freundlich and Langmuir isotherm adsorption model for MB adsorption.

Adsorbent	Freundlich			Langmuir		
	n	K_F_ (L/g)	SSE	q_max_ (mg/g)	K_L_ (L/g)	SSE
RGO 1:1	1.57	8.23	111.73	228.07	0.015	104.41
RGO 1:2	1.49	7.98	227.54	276.06	0.013	213.06
RGO 1:4	1.57	7.29	51.44	209.34	0.014	39.00

Based on the SSE obtained, it can be seen the SSE of the Langmuir model is smaller than the SSE of the Freundlich model. Also, a small *n* value indicates that the adsorbent heterogeneity is minimal and tends to be homogeneous. This shows the uniformity of the adsorption surface that supports conformity with the Langmuir model. The affinity of the adsorption between RGO-MB is determined by the K_L_ parameter where the value is very small; which supports the results of data *n* from the Freundlich model, where small *n* is indicating that the adsorbent more less intensity to adsorb the adsorbate. It can be concluded that the Langmuir isotherm model is the most suitable model for the MB adsorption process using RGO. The process of adsorption of MB by RGO studied tends to be slow.

Table 4 shows the relationship of the variation in concentration to the adsorption capacity. At a ratio of 1:1, the adsorption capacity (q_max_) was found 228.07 mg/g, at a concentration of 1:2 q_max_, the higher adsorption capacity was obtained (276.06 mg/g) and decreased at a ratio of 1:4. This can be possible that the CO group present in the GO has not been completely reduced in the concentration of 1:1, whereas at the ratio of 1:4 the reduced GO is at the saturated condition. The adsorption capacity of MB using various RGO suggests that RGO 1:2 has the best performance to remove MB.

The ability of RGO to adsorb MB was compared to other studied adsorbents, as shown in Table 5. RGO produced in this study exhibited relative higher of adsorption capacity compared to other adsorbents. For the same target adsorbate (MB), up to 276 mg of MB can be adsorbed on the surface of RGO; which is much higher than the adsorption capacity of nanocomposite of β-cyclodextrin/magnetic graphene oxide, that is only 94 mg/g. The photocatalyst Fe_3_O_4_-RGO-TiO_2_, only able to adsorp 1.67 mg MB per gram of particle. However, the use of activated seagrass *Posidonia oceanica* waste can achieve almost ten times higher than our adsorbent^40^. The high adsorption capacity of RGO (this study) can be explained by its high specific surface area, i.e. 3127 m^2^/g as measured by the Brunauer-Emmett-Teller method. The significantly increased of the specific surface area from GO (832 m^2^/g) to RGO has facilitated the MB adsorption. This result suggests that kaffir lime peel extract is potent to be used as the bioreducing agent to modify GO for MB adsorption. Further study to elucidate the mechanism of reduction activity performed by phytochemicals of kaffir lime peels is required.

**Table 5:** Adsorption capacity of several adsorbents to adsorp methylene blue.

Adsorbents	Adsorption capacity (mg/g)	References
Graphene/SrAl_2_O_3_:Bi^3+^	42.92	^17^
β-cyclodextrin/MGO	93.97	^43^
g-C_3_N_4_(Melamine)	1.64	^44^
g-C_3_N_4_(Thiourea)	1.87	^44^
g-C_3_N_4_(Urea)	2.51	^44^
TiO_2_/Na-g-C_3_N_4_	1.8	^45^
Magnetic carboxyl functional nanoporous polymer	57.74	^46^
Activated *Posidonia oceanica* waste	2,681.90	^40^
CeO_2_	4.37	^47^
Fe_3_O_4_-RGO-TiO_2_	1.67	^48^
Ag-Fe_3_O_4_-polydopamine	45	^23^
RGO 1:2	276.06	Present work

### Adsorption thermodynamics

**Figure 6:**
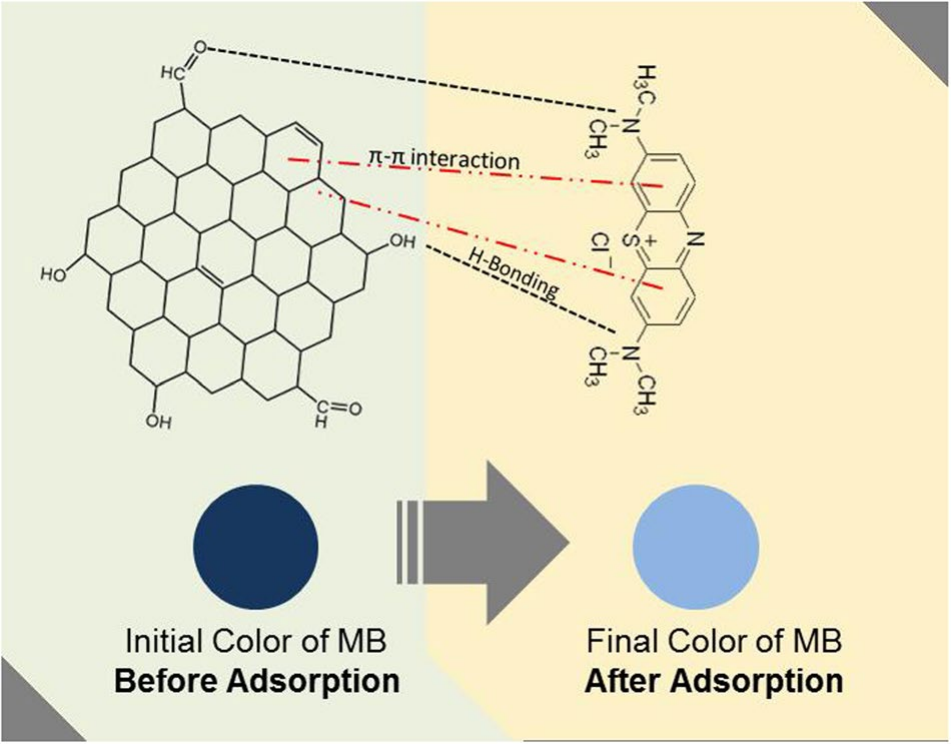
MB adsorption mechanism using RGO.

Adsorption thermodynamics proceeds to evaluate the effect of temperature due to adsorption process of RGO-MB. The thermodynamics parameter is calculated using following equations:

6 $$\Delta {\rm{G}}=-{\rm{RT}}\,\mathrm{ln}\,{K}_{L}$$

7 $$\Delta {\rm{G}}=\Delta {\rm{H}}=\Delta {\rm{S}}.{\rm{T}}$$

8 $$\mathrm{ln}({K}_{L})=\,\mathrm{ln}(\frac{Qe}{Ce})=\frac{\Delta {\rm{S}}}{R}-\frac{\Delta {\rm{H}}}{RT}$$

where R is the universal gas constant (8.314 J mol^−1^ K^−1^) and *T* is absolute temperature (K). ΔH (kJ mol^−1^), ΔS (J mol^−1^ K^−1^), and ΔG (kJ mol^−1^) are enthalpy, entropy, and Gibbs free energy, respectively. ΔH and ΔS were calculated from the slope and intercept of *ln (K*_*L*_) versus *1/T* which was represented in Fig. 5b, and Fig. 5d. As seen in Table 6, the ΔG value was found to be −5.98 kJ mol^−1^ at 303 K and decrease to −8.47 kJ mol^−1^ at 333 K. The negative values of ΔG indicates the MB adsorption onto RGO is spontaneous. The value of ΔH and ΔS was found to be 19.15 kJ mol^−1^ and 82.93 J mol^−1^ K^−1^, respectively. The positive value of ΔH and ΔS can be related to the adsorption mechanism of RGO-MB^41^ as seen in Fig. 6, where the positive value of ΔH also represents that RGO-MB adsorption process was endothermic.

**Table 6:** Thermodynamics parameters for MB adsorption onto RGO.

T (K)	ΔG (kJ mol^−1^)	ΔH (kJ mol^−1^)	ΔS (J mol^−1^ K^−1^)
303	−5.98	19.15	82.93
313	−6.81		
333	−8.47

The high adsorption capacity of RGO is attributed to π-π interaction between MB molecule and the surface of RGO^14^. The high area exhibited by RGO favors the π-π electron donor interaction between the molecule of MB and RGO. Accordingly, the adsorption of MB on the surface of RGO could go up to 276 mg/g. In addition, electrostatic forces caused by difference in surface charge between MB and RGO also support the adsorption process. In aqueous solution, MB will exist as positively charged ions, while RGO tends to be negatively charged in water and thus, this has facilitated the electrostatic force between the two^42^.

## Conclusions

The reduction: of GO to RGO by using kaffir lime peel extract as a bioreductor was successfully carried out. The ratio of bioreductor and reduction time has been observed to influence the characteristics of RGO, and the optimal conditions were found to be 1:2 and 8 h, respectively. Analyses of UV-Vis Spectroscopy, FTIR, and XRD indicated the reduction of GO to RGO by the C=C group restoration. The adsorption study performed on MB showed that the maximum adsorption capacity (q_max_) of RGO was found 276.06 mg/g. MB adsorption by RGO followed pseudo-second-order model; whereas, for the isotherm, the system prefers in line with the Langmuir model. The RGO-MB adsorption interaction is more dominantly controlled by chemisorption. The adsorption of RGO-MB was a spontaneous and endothermic process. The present study indicates RGO prepared by bioreduction of RG using kaffir lime peels extract a potential adsorbent to remove MB and therefore, it can be further developed for industrial applications.
